# Treatment of Chiari Malformation and Concomitant Paediatric Scoliosis Long-Term Follow-Up in One Major Referral Centre in the UK

**DOI:** 10.3390/jcm12103409

**Published:** 2023-05-11

**Authors:** Oded Hershkovich, Raphael Lotan, Netanel Steinberg, Galateia Katzouraki, Daniel D’Aquino, Magnum Tsegaye

**Affiliations:** 1 Centre for Spinal Studies and Surgery, Queen’s Medical Centre, Nottingham NG7 2UH, UK; 2 Department of Orthopedic Surgery, Wolfson Medical Center, Affiliated to the Sackler Faculty of Medicine, Tel Aviv University, Holon 5822012, Israel

**Keywords:** scoliosis, Chiari malformation type 1, curve, progression

## Abstract

Objective: Paediatric scoliosis (PS) and Chiari malformation type 1 (CM-1) have been reported to be associated with each other. Scoliosis curvature is a common finding among patients operated for CM-1, and curve development has been related to it. We report a cohort of PS and CM-1 patients managed with posterior fossa and upper cervical decompression (PFUCD) by a single surgeon, with an average of two years of follow-up. Methods: We present a retrospective cohort in a single referral centre for patients with CM-1 and PS. Results: From 2011 to 2018, we identified fifteen patients with CM-1 and PS; eleven underwent PFUCD, ten had symptomatic CM-1, and one had asymptomatic CM-1 but showed curve progression. The remaining four CM-1 patients were asymptomatic and were hence treated conservatively. The average follow-up post-PFUCD was 26.2 months. Scoliosis surgery was performed in seven cases; six patients underwent PFUCD prior to the scoliosis correction. One scoliosis case underwent surgery in the presence of mild CM-1 treated conservatively. The remaining four cases were scheduled for scoliosis correction surgery, while three were managed conservatively, with one case lost to follow-up. The average time between PFUCD and scoliosis surgery was 11 months. None of the cases had intraoperative neuromonitoring alerts or perioperative neurological complications. Conclusion: CM-1 with concomitant scoliosis can be found. Symptomatic CM-1 might require surgery, but as we discovered, PFUCD had negligible effect on curve progression and the future need for scoliosis surgery.

## 1. Introduction

Adolescent idiopathic scoliosis (AIS) is a common disorder affecting 2–4% of children aged 10–16, and is defined as a lateral curvature exceeding 10 degrees, as measured by the cobb angle, with vertebral rotation on a standing upright radiograph of the spine [1,2,3]. Scoliosis can sometimes be the initial complaint of an underlying brainstem anomaly [4,5]; an atypical scoliotic curve, rapid progression, or neurological impairment should raise suspicion of underlying pathology. An MRI is typically conducted to rule out the organic causes of scoliosis [6]. The incidence of spinal cord or brainstem anomalies in patients diagnosed with AIS was reported from four to fifty-eight percent [7,8,9]. Chiari malformation type I (CM-I) is a tonsillar ectopia that is found in atypical AIS patients. It was first described in 1891 by Chiari as a developmental abnormality of the hindbrain, and is characterised by a downward displacement of the cerebellar tonsils of at least 5 mm below the foramen magnum [10,11]. The definitive surgical intervention for CM-I with or without syringomyelia is posterior fossa upper cervical decompression (PFUCD) with or without duroplasty [12].

The association between scoliosis and CM-I has been widely reported in the literature. The incidence of scoliosis in patients with Chiari malformation and syringomyelia is higher than in the average paediatric population, with a prevalence of up to 13–36% of CM-I patients [4,5,13,14,15]. 

Scoliosis is a common finding among patients operated for CM-1, and curve development has been related to it. Strahle et al. and Brockmeyer et al. reported that for patients with CM-I, syringomyelia, and scoliosis, a younger age at the time of decompression was associated with postsurgical curve improvement, especially for patients younger than ten years of age with curves of 35° or lower [12,16]. Some accept that prior to scoliosis correction surgery, spinal cord or brainstem lesions need to be addressed to prevent potential neurological complications [4,17]. Still, controversy remains regarding the best treatment for patients with CRS (Chiari-related scoliosis) and treatment timing. Over the years, multiple studies have shown that the operative treatment of CM-1 can successfully stop scoliosis progression, or even improve enough to reverse the need for scoliosis correction [13,14,15,18,19,20]. Muhonen et al. reviewed eleven children with CM-1 and scoliosis and found that in eight children, scoliosis improved after PFUCD [14]. Dyste et al. studied eight patients and found an improved curve of scoliosis occurred after decompression with and without surgical drainage of syringomyelia [21]. 

Conversely, several other studies have shown that cervical decompression alone could not treat scoliosis, and further operative treatment was needed. Tubbs et al. and Ghanem et al. reviewed 16 and 12 cases, respectively, and both studies found that decompression alone did not resolve curvatures more than 40° [5,22], while Brockmeyer et al. found less chance of improvement in curves larger than 50° [16]. Isu et al. reported an improvement or preclusion of the scoliotic curve progression in six patients with preoperative angles of less than 40° [23]. Ghanem et al. found a positive correlation between the severity of preoperative structural changes and the absence of spinal deformity improvements after decompression [22]. Other studies have suggested an association is present between early age and a greater chance of scoliosis improvement after decompression [4,14,19]. 

We report a cohort of CM-I and scoliosis patients operated with posterior fossa upper cervical decompression (PFUCD) by a single surgeon, with an average of two years of follow-up. This study aimed to assess the effect of PFUCD on scoliosis curve progression, the need for scoliosis operative treatment, and the safety of scoliosis surgery following PFUCD in CM-I and scoliosis patients.

## 2. Materials and Methods

We conducted a retrospective cohort analysis of patients with CM-1 and paediatric scoliosis (PS). From November 2011 to December 2018, we identified 15 patients with CM-1 and PS. A senior spinal surgeon clinically assessed all the patients. Imaging assessments included PA and lateral standing radiographs for a coronal and sagittal plane curve classification, along with a mandatory cervical-thoracic-lumbar MRI on arrival. Scoliosis patients underwent follow-up radiographs every six months for curvature change until maturity, as ascertained by the Risser score. Surgery for CM-1 was performed on patients suffering from significant symptoms affecting their quality of life, such as headaches or visual disturbances. Asymptomatic CM-1 patients were operated on for scoliotic curve progression. Scoliosis surgery was performed on curves with a Cobb angle of 45 degrees or more, or a curve progression of 10 degrees annually on repeat standing radiographs.

We have conducted a retrospective study based on medical records and images studying our results. The study received a waiver from the ethical committee.

## 3. Results

**Figure 1:**
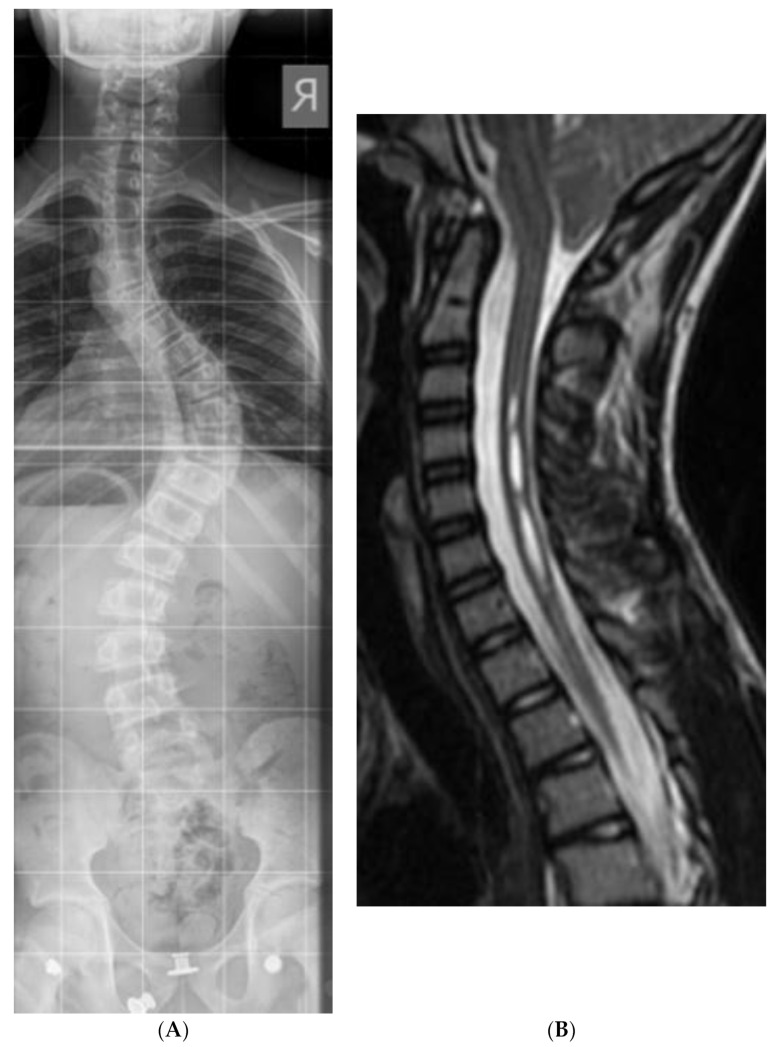
(**A**) Thirteen years old AIS patient with CM-1—AP Spine. (**B**) Thirteen years old AIS patient with CM-1—Cspine MRI.

From November 2011 to December 2018, we identified 15 patients with Chiari malformation type 1 and scoliosis; the average age was 12.7 years (5–19). Scoliosis cases included eleven AIS, two congenital/EOS, and two syndromic (Figure 1A,B). Eleven CM-1 patients were symptomatic; nine of the eleven suffered from headaches, two of the eleven had extremity motor weakness, and four of the eleven suffered headaches and vomiting. The cohort did not suffer from visual disturbances. The symptoms described were considered an indication of posterior fossa decompression. Four were asymptomatic and were followed up. Eleven of the fifteen CM-1 patients had syringomyelia; the other four patients consisted of three with AIS and one with congenital scoliosis. The three AIS patients required scoliosis correction following PFUCD, while the congenital scoliosis was treated conservatively. The average post-operative follow-up was 26.2 months (with a range of 6–59 months). Post-operatively, all patients improved significantly, with two patients reporting a complete resolution of their symptoms. One patient suffered a superficial surgical-site infection that was treated successfully with oral antibiotics. Seven patients underwent scoliosis surgery, and in six of them, PFUCD was performed prior to the scoliosis correction (all were AIS cases). Only one case of scoliosis was operated on in the presence of a mild CM-1 who was treated conservatively (Table 1).

All scoliosis surgeries but one were posterior spinal fusion (PSF); one case underwent anterior spinal release and posterior spinal fusion. Following PFUCD, four patients were scheduled for scoliosis PSF, while three (two congenital and one syndromic scoliosis) were treated conservatively. One case of syndromic scoliosis was lost for follow-up. These four patients that were treated conservatively were found to have significant scoliotic curves without affecting their level of function (wheelchair-bound or bedridden) (Table 1). 

Scoliosis curves were measured before PFUCD, just before scoliosis correction surgery, post-surgery, and at the last follow-up. The average time between PFUCD and scoliosis PSF was 11 months (with a range of 9–18 months). The average pre-operative curves were MT = 76°, L = 50.6°, and post-operatively were MT = 30.4°, L = 24°, respectively. Post-PFUCD, the main thoracic (MT) curves increased by 14.5° on average, and the lumbar curves by 11°, respectively, with one case showing post-PFUCD curve improvement but was deemed not significant to avoid surgery (MT > 50°). No case has reversed the need for scoliosis correction post-PFUCD (Table 2).

## 4. Discussion

Controversy exists regarding the best treatment for patients with CRS (Chiari-related scoliosis) and treatment timing. Over the years, multiple studies have shown that the operative treatment of CM-1 can successfully stop scoliosis progression or even improve enough to reverse the need for scoliosis correction [13,14,15,18,19,20,21,23]. Conversely, several studies showed that decompression alone could not treat scoliosis, especially with curves larger than 40°, and further operative treatment was needed [5,22]. Other studies have suggested an association between early age and a greater chance of scoliosis improvement after decompression [4,14,19]. 

Our study is consistent with the observed correlation between the pre-operative scoliotic curve severity and the absence of post-Chiari-decompression improvement.

Our study’s average pre-PFUCD MT and lumbar curves were 61.5° and 39.6°, respectively, representing curves prone to progress by the literature, thus requiring scoliosis surgery. In our study, these curves showed post-decompression MT and an average lumbar curve progression of 14.5° and 11°, respectively, not precluding the need for post-PFUCD scoliosis surgery. Only one patient showed curve regression, but not sufficiently to avoid surgery. 

The literature is equivocal regarding the safety and outcomes of posterior spinal fusion for deformity correction in patients with CM-1 [4,24,25]. Various neurosurgeons advocate CM-1 decompression prior to scoliosis surgery to reduce the risk of neurological complications. Godzik et al. compared the safety and subjective outcomes of spinal deformity surgery between Chiari malformation type 1 with associated scoliosis patients and a matched AIS cohort. They found a higher rate of neurological deficits (11%) despite adequate decompression and intraoperative neuromonitoring [26,27]. Other studies reported no new neurologic deficits [28]. In our study, seven patients underwent scoliosis surgery, six of whom had PFUCD prior to scoliosis correction. The average interim between PFUCD and scoliosis surgery was 11 months, and no new neurological deficits were sustained, nor were neuro-monitoring abnormalities observed during the surgical correction.

Our study is limited by the retrospective design as well as the small cohort we utilised due to the rarity of the CM-1 and scoliosis combination. As a retrospective design, the clinical data and medical records are limited and not fully available.

## 5. Conclusions

We found PFUCD to be a safe surgery with satisfactory clinical and radiological results, allowing subsequent uneventful scoliosis surgical procedures without neuromonitoring abnormalities or perioperative neurological complications. We also re-validated that PFUCD does not eliminate the need for operative scoliosis correction when indicated, and curve significant curve regression is not to be expected.

Given the limited data available through small sample-sized studies, further research is needed to assess the correlation between the pre-operative scoliosis severity and the chance of spinal deformity regression following PFUCD, and the safety of scoliosis correction surgery in the presence of an un-decompressed CM-1 malformation.

## Figures and Tables

**Table 1:** Demographic characteristics and clinical features of the subjects with Chiari.

	All Patients	PFUCD Operated	SC Operated	SC Pending Surgery	Conservative CM-1	Conservative Scoliosis
Number of patients	15	11	7	4	4	4
Age (on diagnosis of Chiari—year)	12.7	14.2	13	14.75	8.75	10.25
Females (number)	11	8	6	3	3	2
Type of scoliosis (AIS/S/EOS)	11:02:02	10:01	07:00:00	04:00:00	01:01:02	00:02:02
CM-1 (N)	15	11	7	4	4	4
Syrinx (N)	11	9	5	3	2	3
Type of syrinx (cervical/cervico-thoracic/holo-cord)	02:06:03	01:06:02	01:03:01	00:02:01	01:00:01	01:01:01
Symptoms pre-PFUCD (Y)	11	10	6	4	0	1
PFUCD performed (Y)	11	11	6	4	0	1
Average f/u post PFUCD (months)	-	26.2 (6–59)	40.67 (28–59)	8 (6–12)	-	12 (one case)
Average f/u post SC (months)	-	-	34 (10–56)	-	56 (one case)	-

AIS, adolescent idiopathic scoliosis; S, syndromic; EOS, early onset scoliosis/congenital; SC, scoliosis correction; PFUCD, posterior fossa upper cervical decompression; and CM-1, Chiari malformation type 1.

**Table 2:** Curve parameters and measures pre-operative and post-operative.

Curve Parameters and Measures:	Average Degrees
Pre PFUCD surgery thoracic Cobb (Average Angle)	61.5
Pre SC surgery thoracic Cobb (Average Angle)	76.0
Pre PFUCD surgery lumbar Cobb (Average Angle)	39.6
Pre SC surgery lumbar Cobb (Average Angle)	50.6
Change in MT curve from PFUCD to SC	14.5
Change in L curve from PFUCD to SC	11.0
Average waiting time from PFUCD to SC (months)	11.0

MT curve, main thoracic curve; L curve, lumbar curve; SC, scoliosis correction; and PFUCD, posterior fossa upper cervical decompression.

## Data Availability

The complete data are available under a confidentiality restriction.
